# Differentiation of Acute Internal Carotid Artery Occlusion Etiology on Computed Tomography Angiography: Diagnostic Tree for Preparing Endovascular Treatment

**DOI:** 10.3390/diagnostics14141524

**Published:** 2024-07-15

**Authors:** Bo Kyu Kim, Byungjun Kim, Sung-Hye You

**Affiliations:** Department of Radiology, Korea University Anam Hospital, Korea University College of Medicine, 73 Inchon-ro, Seongbuk-gu, Seoul 02841, Republic of Korea; stingray0379@naver.com (B.K.K.); yshneuro@gmail.com (S.-H.Y.)

**Keywords:** acute ischemic stroke, CT angiography, internal carotid artery, endovascular treatment

## Abstract

Background and Purpose: This study aimed to identify the imaging characteristics and discriminate the etiology of acute internal carotid artery occlusion (ICAO) on computed tomography angiography (CTA) in patients with acute ischemic stroke. Materials and Methods: We retrospectively evaluated consecutive patients who underwent endovascular thrombectomy for acute ICAO. Contrast filling of the extracranial ICA in preprocedural CTA was considered apparent ICAO. Non-contrast filling of the extracranial ICA was evaluated according to the contrast-filled lumen configuration, lumen margin and location, Hounsfield units of the non-attenuating segment, and presence of calcification or an intimal flap. Digital subtraction angiography findings were the reference standard for ICAO etiology and the occlusion site. A diagnostic tree was derived using significant variables according to pseudo-occlusion, atherosclerotic vascular disease (ASVD), thrombotic occlusion, and dissection. Results: A total of 114 patients showed apparent ICAO (*n* = 21), pseudo-occlusion (*n* = 51), ASVD (*n* = 27), thrombotic occlusion (*n* = 9), or dissection (*n* = 6). Most pseudo-occlusions (50/51, 98.0%) showed dependent locations with ill-defined contrast column margins and classic flame or beak shapes. The most common occlusion site of pseudo-occlusion was the petro-cavernous ICA (*n* = 32, 62.7%). Apparent ICAO mainly appeared in cases with occlusion distal to the posterior communicating artery orifice. ASVD showed beak or blunt shapes in the presence of low-density plaques or dense calcifications. Dissection revealed flame- or beak-shaped appearances with circumscribed margins. Thrombotic occlusions tended to appear blunt-shaped. The decision-tree model showed a 92.5% overall accuracy. Conclusions: CTA characteristics may help diagnose ICAO etiology. We provide a simple and easy decision-making model to inform endovascular thrombectomy.

## 1. Introduction

Acute ischemic stroke (AIS) with internal carotid artery occlusion (ICAO) is typically associated with large territorial infarction and poor collaterals, resulting in poor clinical outcomes and high mortality rates [1,2]. Owing to the poor response to intravenous tissue plasminogen activator (IV tPA), the current management of acute ICAO focuses on endovascular thrombectomy (EVT) for the rapid recanalization and restoration of cerebral blood flow [3,4,5,6].

ICAO can occur over a wide range, from the cervical ICA to the ICA terminus, and can show a wide imaging spectrum depending on the thrombus extent and occlusion etiology [7]. Computed tomography angiography (CTA) is a widely used and reliable imaging modality for diagnosing ICAO [8]. However, CTA may not accurately reflect the actual site of occlusion. In particular, the non-contrast filling of the extracranial ICA does not always indicate true occlusion. This phenomenon, called pseudo-occlusion, occurs because CTA acquisition is faster than the rate at which the contrast fills the ICA and can be observed in cases of distal ICA occlusion [9,10]. Approximately 6–15% of patients with acute ischemic stroke show pseudo-occlusion on CTA [9,11,12,13].

The EVT strategy differs according to the thrombus location and extent; it also depends on the occlusion etiology. Patients with atherosclerotic plaques may require angioplasty or extracranial carotid artery stenting. Identifying the true lumen during the procedure is crucial in patients with carotid artery dissection. Occasionally, several aspirations using a large-bore aspiration catheter may be necessary in cases with a large burden of thrombus filling the extracranial ICA. Therefore, predicting the etiology of ICAO on preprocedural CTA is essential for planning the procedure and may be related to shortening the recanalization time and improving the prognosis [6].

Among the imaging findings suggesting the etiology of carotid occlusion, the configuration of the contrast-filled lumen on CTA may help discriminate pseudo-occlusion from true occlusion [14,15,16]. However, obtaining an accurate diagnosis is difficult, possibly because of the low sensitivity of imaging characteristics and low interobserver agreement [12,13,17]. In addition, information on CTA classification for etiologies of true occlusion is limited. Therefore, this study aimed to identify the imaging characteristics and determine the etiology of acute ICAO in patients who underwent EVT.

## 2. Methods

### 2.1. Patient Selection

This retrospective study was approved by the institutional review board, and the requirement for informed consent was waived. We reviewed 336 consecutive patients who underwent EVT for acute large vessel occlusion (LVO) in a prospectively collected single-center database between January 2018 and December 2022. The inclusion criteria were (1) acute LVO in the anterior circulation; (2) available preprocedural CTA data; and (3) no contrast filling of the ICA on CTA.

### 2.2. Imaging Protocol

CTA was performed using a 128-channel multi-detector CT scanner (Somatom Definition AS+; Siemens, Erlangen, Germany). The scanner was set to 100 kV, 200 mAs, with a pitch of 1.2, rotation time of 0.5 s, and table speed of 92 mm/s for CTA. A total of 90 mL of contrast agent (iohexol, 350 mg iodine/mL) was injected intravenously at a rate of 5.0 mL/s, followed by 30 mL of saline flushing at a rate of 5.0 mL/s. Bolus triggering was performed at the ascending aorta with a threshold of 100 Hounsfield units. Caudocranial acquisition from the aortic arch to the skull vertex began when the Hounsfield units of the aortic arch reached the settled threshold to acquire arterial-phase CTA.

### 2.3. CTA Analysis

Two neurointerventionists blinded to the digital subtraction angiography (DSA) findings independently reviewed the CTA images, and discrepancies were resolved by consensus. On CTA, 1 mm axial, coronal, sagittal, and maximum-intensity projection images were reviewed together. Contrast filling of the cervical ICA was classified as apparent ICAO. When non-contrast filling was observed in the extracranial ICA, the contrast column of the rudimentary lumen was evaluated as follows: according to Prakkamakul et al., the shape of the rudimentary lumen of an extracranial ICA can be classified as flame, beak, or blunt [12]. The flame shape is defined as an elongated tapering of the extracranial ICA lumen in which the point of contrast disappearance occurred >2 cm above the carotid bifurcation. In the beak shape, the tapering of the ICA column ends in the coronal or sagittal image; however, the endpoint occurs within 2 cm of the carotid bifurcation. In the blunt shape, an abrupt or flat end of the ICA column occurs within 2 cm of the carotid bifurcation. In this study, the location and margin of the rudimentary lumen were evaluated on the axial image at the level of the cranial location at which the most contrast filling was visible. The location of the lumen was classified as dependent or non-dependent, and the margin was classified as circumscribed or ill-defined. In blunt-shaped lumens, the location was classified as non-dependent. The Hounsfield unit of the non-contrast filling portion was evaluated using the same axial image. The presence of vessel wall calcification at the proximal ICA was also evaluated and classified as mild (punctate calcification), moderate (linear or confluent calcification without intraluminal protrusion), or severe (any calcification protruding into the lumen). Furthermore, we investigated whether a curvilinear hypoattenuated stripe, suspected to be an intimal flap, was present in the carotid bulb or the proximal ICA (Supplementary Figures S1–S6).

### 2.4. DSA Analysis

DSA findings and intraprocedural records were used as reference standards for ICAO etiology. In all patients, the occlusion site was confirmed using delayed angiography at the ipsilateral CCA or microcatheter angiography at the distal segment of the cervical ICA.

**Figure 1:**
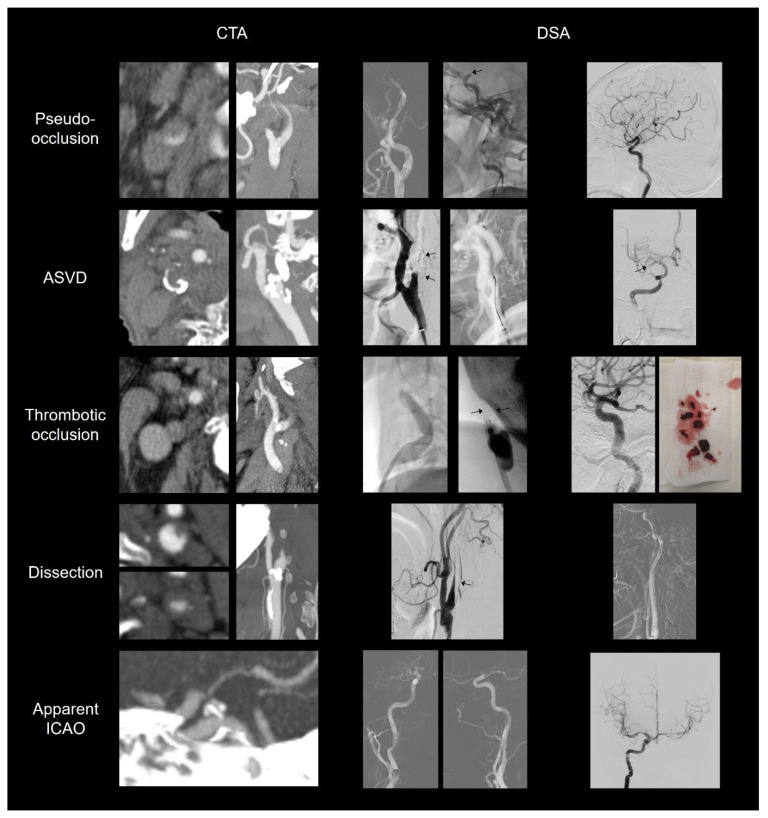
Representative computed tomography angiography (CTA) images and digital subtraction angiography (DSA) findings of internal carotid artery occlusion (ICAO) etiologies. **First row**: A patient with ICA terminus occlusion shows non-contrast filling of the left cervical ICA with a flame shape. In the axial image, the contrast is in the dependent portion, with an ill-defined margin. On DSA, delayed angiogram confirms the occlusion site at the ophthalmic ICA (arrow). After recanalization, the ICA shows no underlying stenosis. **Second row**: CTA shows ICAO due to a low-density plaque with dense calcification. The DSA image shows a blunt-shaped ICA occlusion. The wire had difficulty passing through the occluded segment. After carotid stenting, the aspiration catheter could pass through the stent and navigate to the intracranial tandem occlusion present in the middle cerebral artery (arrow). **Third row**: Images of a patient with ICA terminus occlusion with a blunt-shaped proximal ICA. On DSA, the occlusion started from the mid-cervical ICA. Upon contact aspiration using a large-bore aspiration catheter, a thrombus was retrieved while attached to the catheter tip (arrows). Multiple uses of contact aspiration resulted in the successful removal of a large amount of thrombus filling from the mid-cervical ICA to the ICA terminus. **Fourth row**: Images of a patient with ICA terminus occlusion. The CTA image shows a beak shape and circumscribed margin of cervical ICA. The common carotid angiogram shows a flame-shaped appearance with an intimal flap. The false lumen with stagnated contrast media is located posteriorly (arrow). After placing the guiding catheter in the true lumen, the roadmap image shows a dissecting flap and intracranial tandem occlusion. **Bottom row**: Images of a patient with apparent ICAO. CTA and DSA show ICA occlusion distal to the posterior communicating artery orifice. The contrast reaches the intracranial ICA and fills the ophthalmic and posterior communicating arteries.

DSA records were comprehensively evaluated for the following findings: (1) delayed contrast filling or contrast stagnation at the cervical ICA; (2) any resistance to microcatheter or microwire exploration through the cervical ICA; (3) the presence of retrograde contrast filling on microcatheter angiography; and (4) the presence and amount of aspirated thrombus by contact or remote aspiration at the proximal ICA. We classified the extracranial ICAO etiology into four categories: pseudo-occlusion, atherosclerotic vascular disease (ASVD), thrombotic occlusion, and dissection. Pseudo-occlusion refers to the absence of an intramural thrombus or in situ stenosis in the extracranial ICA. ASVD refers to proximal ICA stenosis caused by atherosclerotic plaques requiring treatment (e.g., balloon angioplasty with or without stenting). When DSA confirmed only low-grade stenosis, the case was regarded as a pseudo-occlusion. Thrombotic occlusion refers to a long-segment thrombus filling both the intracranial and extracranial ICA, which is aspirated using contact or remote aspiration at the proximal ICA. Carotid dissection was diagnosed based on a classic appearance on DSA, a pseudo-lumen or an intimal flap confirmed on microcatheter angiography, and the involvement of the level above the carotid bulb. Figure 1 shows representative CTA and DSA findings for each etiology.

### 2.5. Statistical Analysis

Statistical analyses were performed using IBM SPSS Statistics for Windows, version 22.0 (IBM Corp., Armonk, NY, USA). Because the apparent ICAO could be distinguished by CTA, a statistical analysis was performed among the four groups with non-contrast filling of the extracranial ICA. *p* < 0.05 was considered statistically significant. All variables were compared among the four groups using Pearson’s chi-square or Fisher’s exacts test for categorical variables and one-way ANOVA with post hoc analysis with Bonferroni corrections for continuous variables. Statistically significant variables were used for the decision tree analysis. In the decision tree analysis, the chi-square automatic interaction detection model was used for the differential diagnosis of the etiology of ICA occlusion (minimum cases in the parent node, 9; minimum cases in the child node, 4; maximum tree depth, 5). Because the shape of the rudimentary lumen is the most widely used clue for diagnosis, we used it as the first variable. Cohen’s Kappa value was measured to determine inter-reader agreement between two raters.

## 3. Results

**Figure 2:**
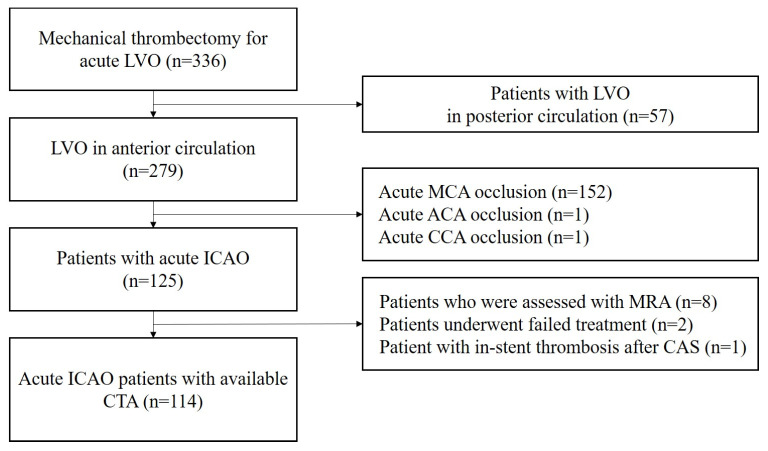
Flowchart of the patients included in this study. ACA, anterior cerebral artery; CAS, carotid artery stenting; CCA, common carotid artery; CTA, computed tomography angiography; ICA, internal carotid artery; LVO, large vessel occlusion; MCA, middle cerebral artery; MRA, magnetic resonance angiography.

Of the 336 patients treated with EVT, 279 had an LVO in the anterior circulation. ICAO was observed in 125 patients. Eight patients underwent magnetic resonance angiography as an initial evaluation, and two patients with failed treatment and whose etiology of occlusion could not be confirmed were excluded. One patient was excluded because the cause of the ICAO was the re-occlusion of previous carotid artery stenting. Ultimately, this study included 114 patients. Figure 2 shows the patient selection process. The number of patients according to etiology was as follows: apparent ICAO (*n* = 21), pseudo-occlusion (*n* = 51), ASVD (*n* = 27), thrombotic occlusion (*n* = 9), and dissection (*n* = 6).

**Figure 3:**
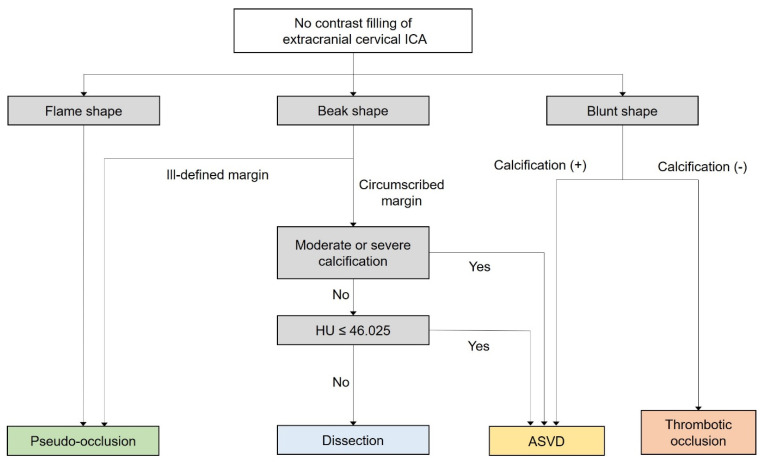
Diagnostic tree for the differential diagnosis of cervical internal carotid artery non-attenuation. ASVD, atherosclerotic vascular disease; ICA, internal carotid artery; HU, Hounsfield unit.

**Figure 4:**
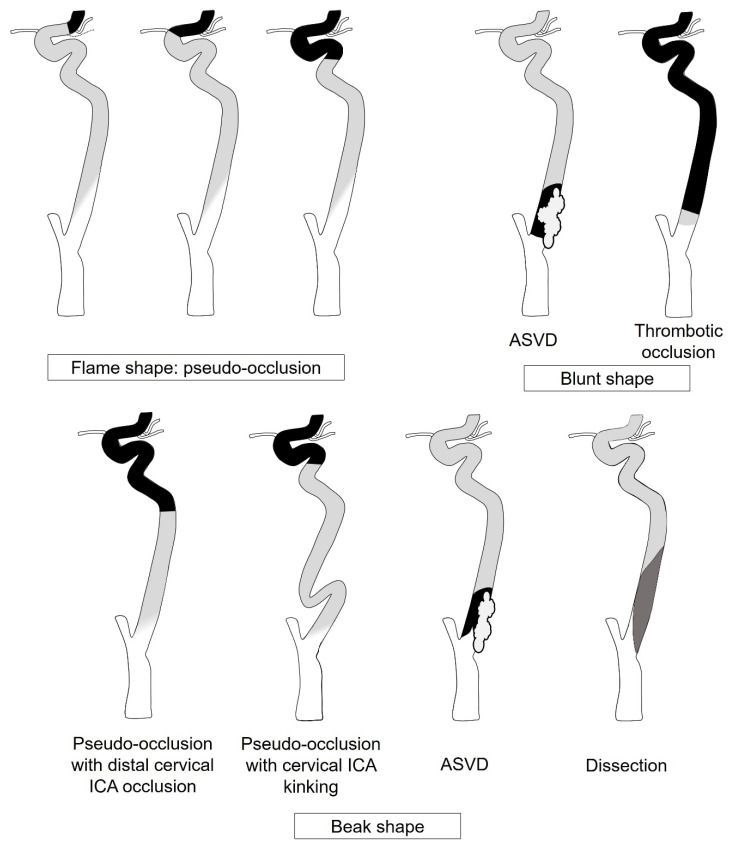
Pathologies of ICA occlusion according to the rudimentary column shape. ASVD, atherosclerotic vascular disease.

Overall, the inter-reader agreement was very good (Supplementary Table S1). Table 1 summarizes the CTA findings according to ICAO etiology. A flame shape was observed in 45 of 51 (88.2%) patients with pseudo-occlusion. Moreover, 50 of 51 (98.0%) patients with pseudo-occlusion had a dependent location of the rudimentary lumen and an ill-defined margin. All patients with ASVD showed a beak shape with a non-dependent location or a blunt shape. Severe calcification protruding into the vessel lumen was observed only in patients with ASVD. Thrombotic occlusion tended to have a blunt shape (77.8%), and the incidence of accompanying calcification was lower than that of ASVD (33.3% vs. 85.2%, *p* < 0.001). Five patients (83.3%) with carotid dissection showed a beak shape with circumscribed margins, and the lumen was located on the non-dependent portion in the axial image. The Hounsfield units of the non-contrast filling segment were higher in pseudo-occlusion than in ASVD (*p* < 0.001); however, the remaining two groups showed no significant differences (Supplementary Figure S7). Figure 3 shows the diagnostic tree analysis based on the imaging characteristics, and full model information is shown in Supplementary Figure S8. The overall accuracy of the model was 92.5%. Figure 4 shows a schematic diagram of the pathologies to consider when ICAO is present, based on the shape of the contrast column.

The actual occlusion sites in patients with pseudo-occlusion are summarized in Table 2. The petro-cavernous internal carotid artery was the most common site of carotid occlusion (62.7%). In four patients, DSA confirmed the maintenance of the antegrade flow of the extracranial ICA, which was considered a partial recanalization of the ICA after IV tPA injection. The occlusion site was more proximal in patients with pseudo-occlusion than in those with apparent ICAO, who had occlusions mainly in the communicating ICA (61.9%).

## 4. Discussion

The main finding of our study was that preprocedural CTA for acute ICAO suggests the etiology of the occlusion with discernible imaging findings. In addition, the frequency of apparent ICAO in acute ICAO was low (18.4%), and non-contrast filling of the extracranial ICA was a common CTA finding in patients with acute ICAO. Among patients with non-contrast filling of the extracranial ICA, 54.8% showed a patent extracranial ICA, and the most common occlusion site was the petro-cavernous ICA.

Pseudo-occlusion is a well-known phenomenon that is accompanied by distal ICAO [11,12,13]. More specifically, it occurs when most of the large branches of the ICA that act as outlets of blood flow are obstructed; thus, the ICA becomes a long-segment blind pouch, and the stagnated blood interferes with contrast inflow. This shows a flame-shaped appearance on both CTA and DSA. This flame-shaped appearance is a sign of high specificity for diagnosing pseudo-occlusions [12]. In our study, all patients with petro-cavernous ICA occlusions showed a typical flame-shaped contrast column on CTA. When the occlusion site was distal to the posterior communicating artery, the contrast reached the intracranial ICA and was diagnosed as an apparent ICAO. The ophthalmic artery was considered to play a role in blood outflow in only approximately half of the patients. In our study, we observed flame-shaped pseudo-occlusion in 5 of the 12 patients (41.7%) with ophthalmic ICA occlusion.

If the occlusion site was more proximal, the length of the contrast column was shorter. Patients with occlusion of the distal cervical ICA or kinking of the mid-cervical ICA showed a beak shape rather than a flame shape. However, unlike true occlusions, the contrast column in these patients was located in the dependent portion and exhibited an ill-defined margin (Supplementary Figure S9). In pseudo-occlusion, since the iodine contrast has a higher specific gravity than blood, the contrast is in the dependent portion of the patient ICA, and non-opacified blood is in the non-dependent portion. Because no structure separates the two components, they can partially mix and form an ill-defined margin on CTA. Furthermore, the Hounsfield units of the non-opacified lumen were higher than those of the other etiologies.

In the present study, the carotid dissection of five of six patients (83.3%) showed a beak-shaped appearance, and all patients demonstrated circumscribed margins. In the presence of complete occlusion due to carotid dissection, the typical string sign or flame or rat-tail sign may not be prominent unlike DSA (representative case in Figure 1). Furthermore, a curvilinear hypoattenuated stripe is not a reliable finding for diagnosing an intimal flap. This was presumed to be due to pseudo-dissection, which is a flow-related artifact [10,18]. An intimal flap could barely be detected in cases of complete occlusion without contrast filling in the true lumen. Meanwhile, the false and true lumens were separated by the intimal flap, implying a circumscribed margin. A previous study also suggested the contrast margin as a possible factor for discriminating dissection [13]. Furthermore, a crescent lumen shape in axial images can be helpful in diagnosis [15].

ASVD can be easily distinguished when CTA shows a low Hounsfield unit and a circumscribed margin by a low-density plaque with a lipid core. Dense calcified plaques also suggest ASVD. However, punctate calcification can often be present without considerable luminal narrowing; therefore, caution is needed when diagnosing ASVD based solely on calcification.

In previous studies, a large thrombus filling the ICA, occasionally accompanied by cardioembolism, was rarely considered as a differential diagnosis in CTA [1,12,13,17]. Since pseudo-occlusion was defined only with the absence of the pathology of proximal ICA, these findings were likely classified as pseudo-occlusion. This may be the cause of the low specificity of the flame-shaped appearance in pseudo-occlusion diagnosis. When the thrombus propagates to the extracranial ICA, the rudimentary column is absent or very short; therefore, most cases show blunt or short-beaked shapes with non-dependent locations.

The results of this study may help establish EVT strategies for patients with acute ischemic stroke. The procedure should be considered depending on the necessity of carotid artery stenting or the estimated thrombus length. In particular, in cases with a large thrombus burden, a large-bore aspiration catheter or even a balloon-guiding catheter may be obstructed by the thrombus. Therefore, a guiding system to bypass the aortic arch is required to eliminate the unnecessary re-selection of the carotid artery and reduce the procedure time. If CTA reveals no typical pseudo-occlusion or ASVD findings, thrombotic occlusion or dissection should be suspected, which may lead to successful recanalization.

Our study has some limitations owing to its retrospective design and modest sample size. The number of patients with a specific etiology (e.g., dissection) was too small to draw conclusions; therefore, further data are required. Second, spontaneous recanalization or changes in the carotid shape may have resulted from IV tPA administration before EVT. However, considering the low response rate to IV tPA in the presence of ICAO and the short interval between CTA and DSA (average, 90 min), this conclusion is unlikely to change [19]. Finally, the scan timing of single-phase CTA may influence the configuration of the ICA, particularly in pseudo-occlusion. The optimal threshold of bolus triggering or CT table speed for patients with hyperacute stroke has yet to be established; however, additional late-phase angiography or four-dimensional CTA may further increase the diagnostic performance of pseudo-occlusion [20].

## 5. Conclusions

CTA findings help differentiate the etiologies of ICAO in patients with AIS. The combination of ICA configuration with rudimentary lumen characteristics may further increase the predictive value of the diagnosis.

## Figures and Tables

**Table 1:** Comparison of the imaging characteristics of each carotid artery occlusion etiology.

Diagnosis	Pseudo-Occlusion (*n* = 51)	ASVD (*n* = 27)	Thrombotic Occlusion (*n* = 9)	Dissection (*n* = 6)	Apparent ICAO (*n* = 21)	*p*-Value
Shape						<0.001
Flame	45 (88.2%)	0 (0%)	0 (0%)	1 (16.7%)		
Beak	6 (11.8%)	17 (63.0%)	2 (22.2%)	5 (83.3%)		
Blunt	0 (0%)	10 (37.0%)	7 (77.8%)	0 (0%)		
Tubular					21 (100%)	
Location						<0.001
Dependent	50 (98.0%)	0 (0.0%)	0 (0.0%)	1 (16.7%)	n.a.	
Non-dependent	1 (2.0%)	27 (100%)	9 (100.0%)	5 (83.3%)	n.a.	
Margin						<0.001
Ill-defined	50 (98.0%)	2 (7.4%)	3 (33.3%)	1 (16.7%)	n.a.	
Circumscribed	1 (2.0%)	25 (92.6%)	6 (66.7%)	5 (83.3%)	n.a.	
Hounsfield Unit	117.6 ± 24.0	59.3 ± 22.6	84.7 ± 32.6	88.4 ± 30.9	n.a.	<0.001
Calcification						<0.001
Present	10 (19.6%)	23 (85.2%)	3 (33.3%)	4 (66.7%)	n.a.	
Absent	41 (81.4%)	4 (14.8%)	6 (66.7%)	2 (33.3%)	n.a.	
Degree of calcification						<0.001
Mild	4 (7.8%)	4 (14.8%)	0 (0.0%)	4 (66.7%)	n.a.	
Moderate	6 (11.8%)	7 (25.9%)	3 (33.3%)	0 (0.0%)	n.a.	
Severe	0 (0.0%)	12 (44.4%)	0 (0.0%)	0 (0.0%)	n.a.	
Curvilinear hypoattenuated stripe						0.096
Present	6 (11.8%)	1 (3.7%)	1 (11.1%)	1 (16.7%)	n.a.	
Absent	45 (88.2%)	26 (96.3%)	8 (88.9%)	5 (83.3%)	n.a.	

Data are expressed as number (%) of patients for nominal variables and mean ± standard deviation for the continuous variable. ASVD, atherosclerotic vascular disease; ICAO, internal carotid artery occlusion; n.a., not accessed.

**Table 2:** Occlusion site confirmed on DSA in patients with pseudo-occlusion and apparent ICAO.

Etiology	Occlusion Site on DSA	ICA Shape on CTA	Number	Remarks
Pseudo- occlusion (*n* = 51)	Distal cervical ICA	Beak	5 (9.8%)	
	Petro-cavernous ICA	Flame	32 (62.7%)	
		Beak	1 (2.0%)	Kinking of proximal cervical ICA
	Ophthalmic ICA	Flame	5 (9.8%)	
	Communicating ICA	Flame	3 (5.9%)	Spontaneous recanalization after IV tPA injection (2/3)
				Hypoplastic posterior communicating artery (1/3)
	Terminal ICA	Flame	1 (2.0%)	Spontaneous recanalization after IV tPA injection
	MCA	Flame	4 (7.8%)	Spontaneous recanalization after IV tPA injection
Apparent ICAO (*n* = 21)	Ophthalmic ICA		7 (33.3%)	
	Communicating ICA		13 (61.9%)	
	Terminal ICA		1 (4.8%)	

CTA, computed tomography angiography; DSA, digital subtraction angiography; ICA, internal carotid artery; ICAO, internal carotid artery occlusion; IV tPA, intravenous tissue plasminogen activator; MCA, middle cerebral artery.

## Data Availability

All data relevant to the study are included in the article or uploaded as Supplementary Materials.

## Supplementary Materials

The following supporting information can be downloaded at: https://www.mdpi.com/article/10.3390/diagnostics14141524/s1, Figure S1: Shape; Figure S2: Location; Figure S3: Margin; Figure S4: HU; Figure S5: Calcification; Figure S6: Hypoattenuated stripe; Figure S7: Post-hoc analysis; Figure S8: Diagnostic tree; Figure S9: Kinking case (a–e); Table S1: Interreader agreements for computed tomography findings.

## Abbreviations

AIS = acute ischemic stroke; ASVD = atherosclerotic vascular disease; CTA = computed tomography angiography; DSA = digital subtraction angiography; EVT = endovascular thrombectomy; ICAO = internal carotid artery occlusion; IV tPA = intravenous tissue plasminogen activator; LVO = large vessel occlusion.
