# Supplementary material for: Differentiation of Acute Internal Carotid Artery Occlusion Etiology on Computed Tomography Angiography: Diagnostic Tree for Preparing Endovascular Treatment
Source: Diagnostics (Basel). 2024 Jul 15;14(14):1524. doi: 10.3390/diagnostics14141524 (PMC11276486; doi:10.3390/diagnostics14141524)
Supplement: Supplementary file 1 [file diagnostics-14-01524-s001.zip › Table S1.pdf]

Table S1. Interreader agreements for computed tomography findings.

CT findings	Kappa	95% CI
Shape	0.792	0904 – 0.680.
Location	0.914	0.996 – 0.832
Margin	0.820	0.940 – 0.700
Degree of calcification	0.913	0.986 – 0.840
Curvilinear hypoattenuated stripe	0.839	1.000 – 0.663
