# Supplementary figures and images for: Differentiation of Acute Internal Carotid Artery Occlusion Etiology on Computed Tomography Angiography: Diagnostic Tree for Preparing Endovascular Treatment
Source: Diagnostics (Basel). 2024 Jul 15;14(14):1524. doi: 10.3390/diagnostics14141524 (PMC11276486; doi:10.3390/diagnostics14141524)

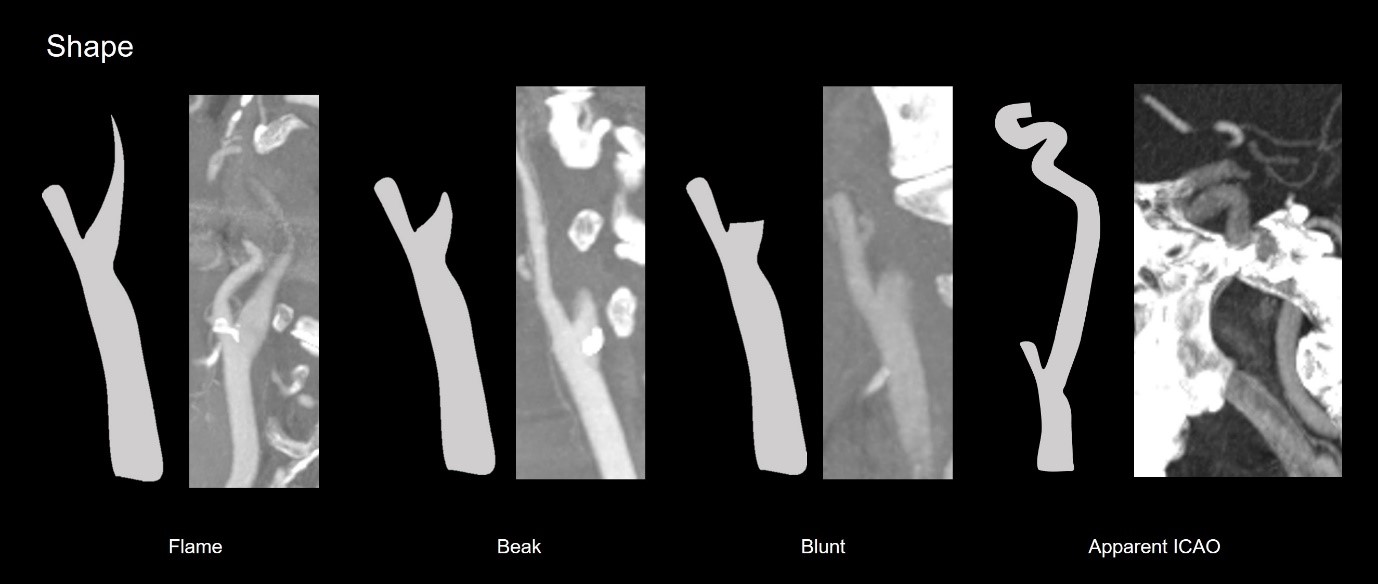

Supplement: Supplementary file 1 [file diagnostics-14-01524-s001.zip › Figure S1_Shape.jpg]

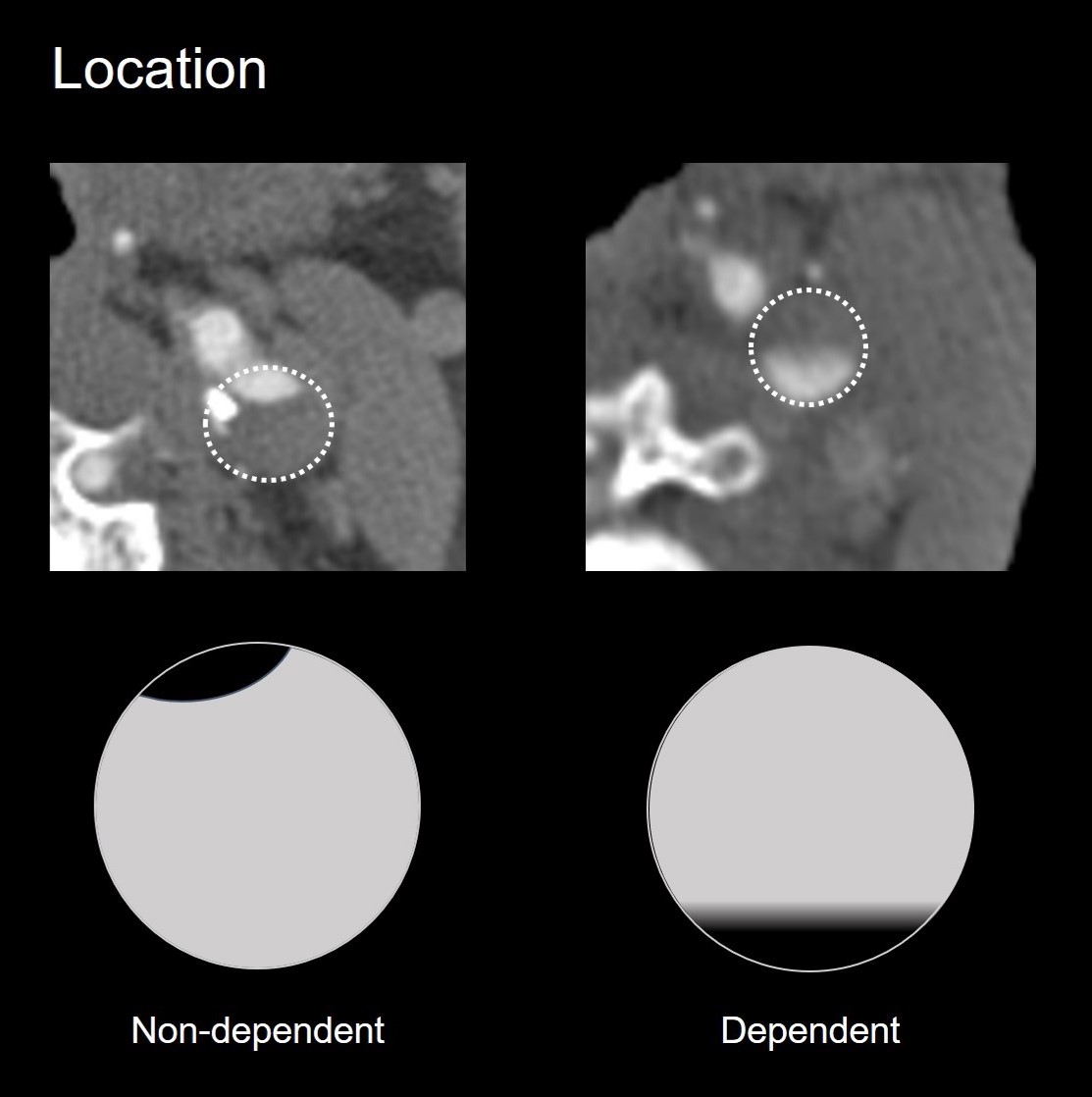

Supplement: Supplementary file 1 [file diagnostics-14-01524-s001.zip › Figure S2_Location.jpg]

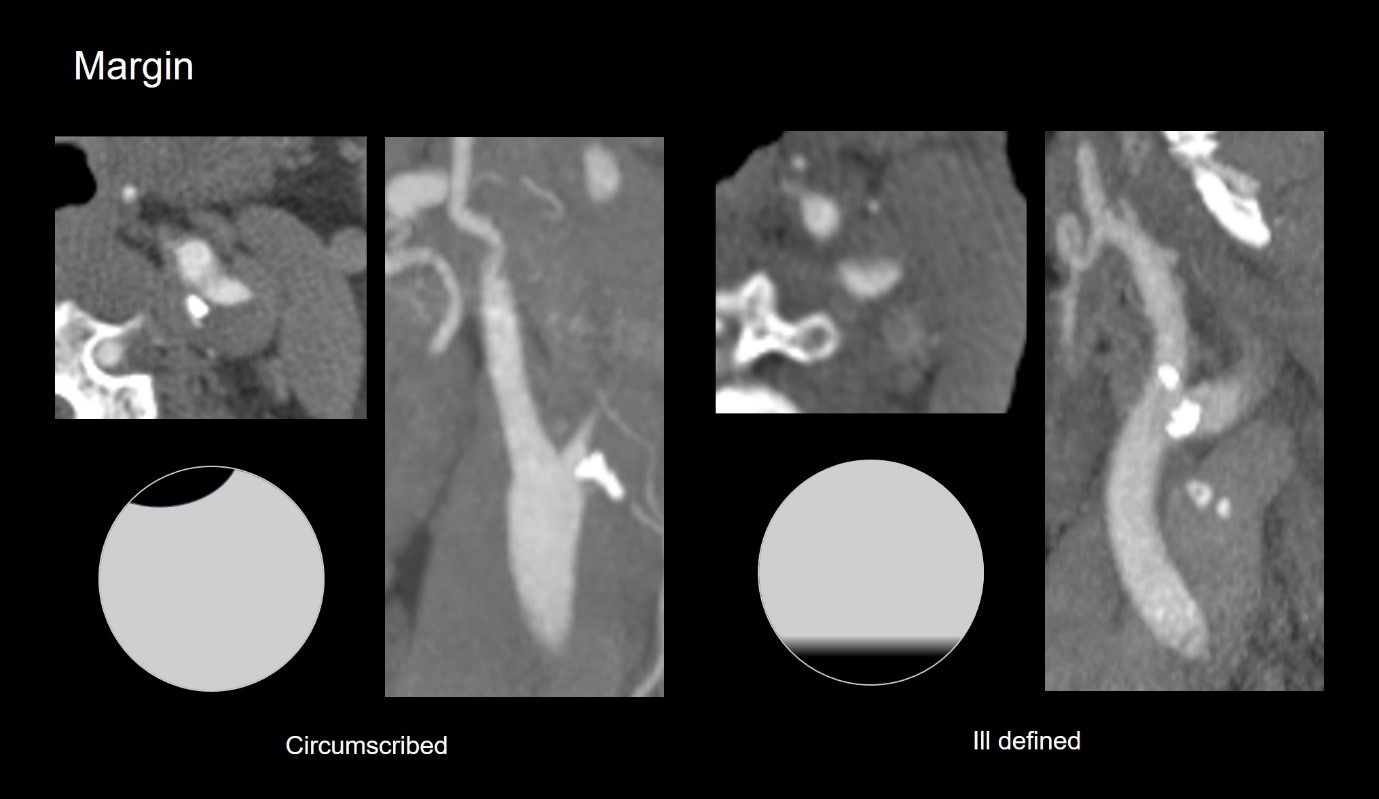

Supplement: Supplementary file 1 [file diagnostics-14-01524-s001.zip › Figure S3_Margin.jpg]

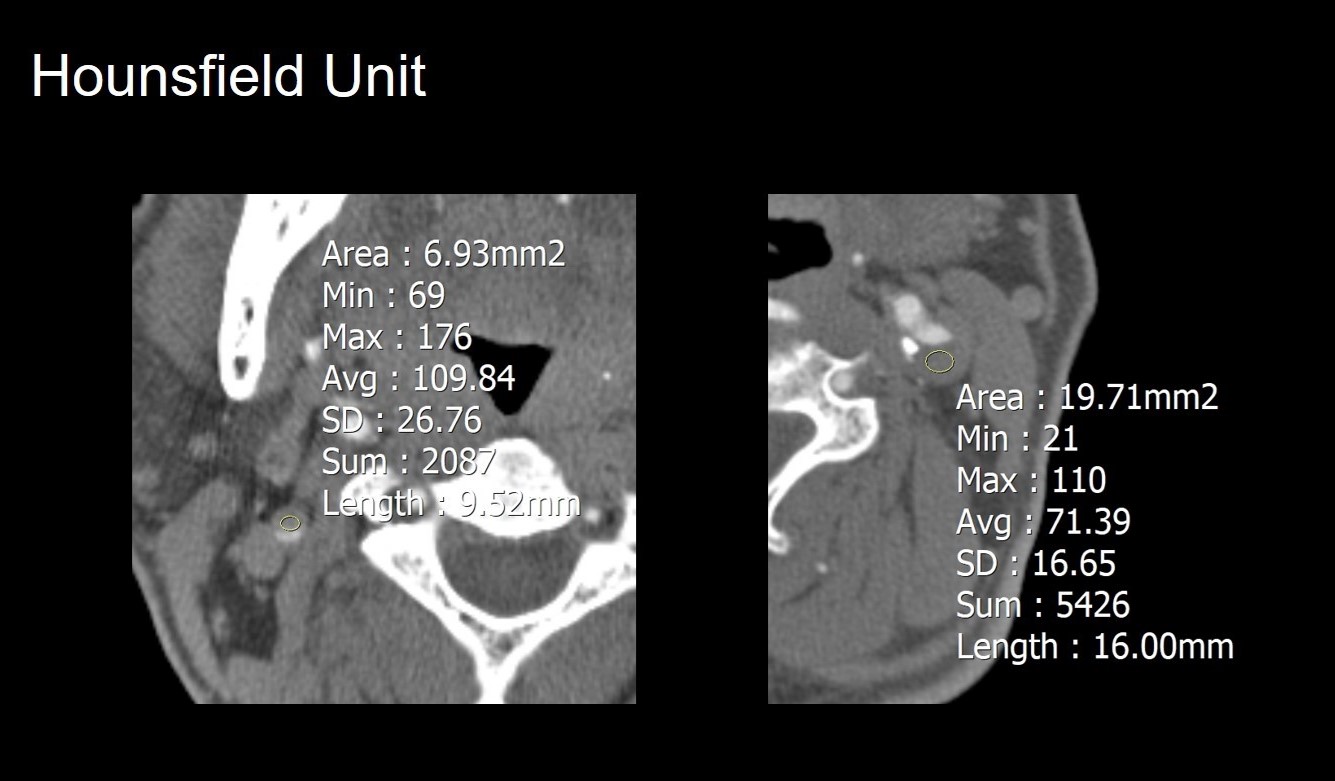

Supplement: Supplementary file 1 [file diagnostics-14-01524-s001.zip › Figure S4_Hu.jpg]

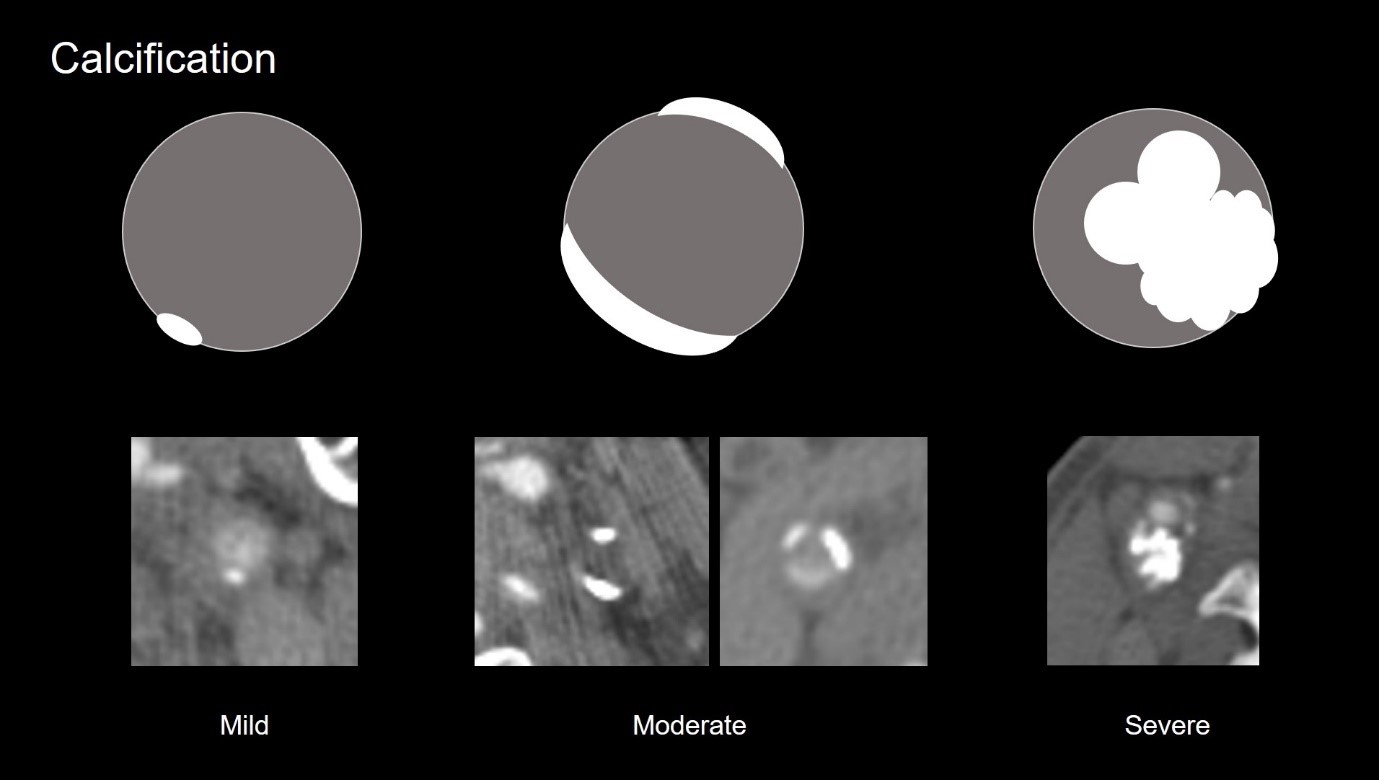

Supplement: Supplementary file 1 [file diagnostics-14-01524-s001.zip › Figure S5_Calcification.jpg]

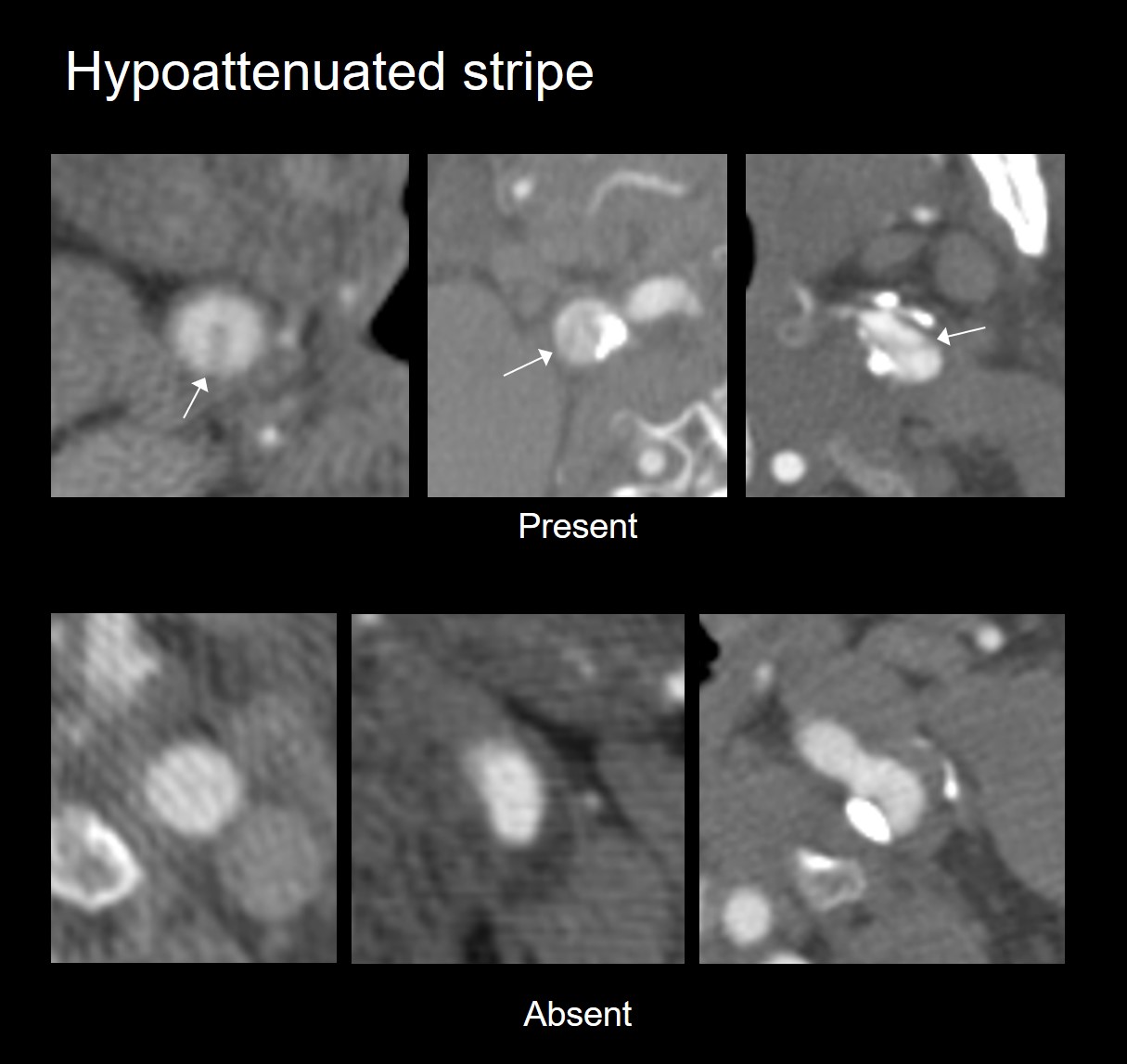

Supplement: Supplementary file 1 [file diagnostics-14-01524-s001.zip › Figure S6_Hypoattenuated stripe.jpg]

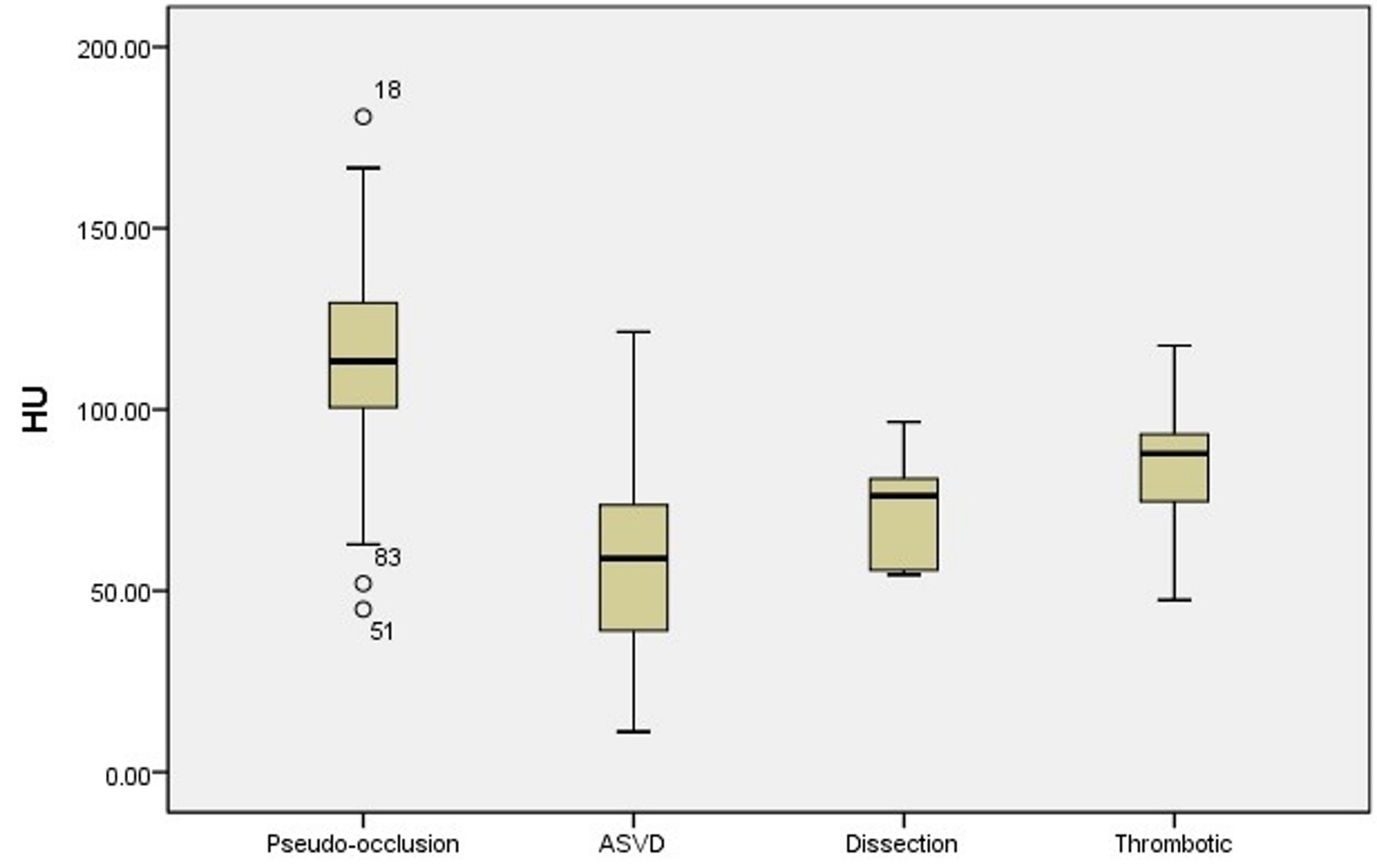

Supplement: Supplementary file 1 [file diagnostics-14-01524-s001.zip › Figure S7_Post-hoc analysis.jpg]

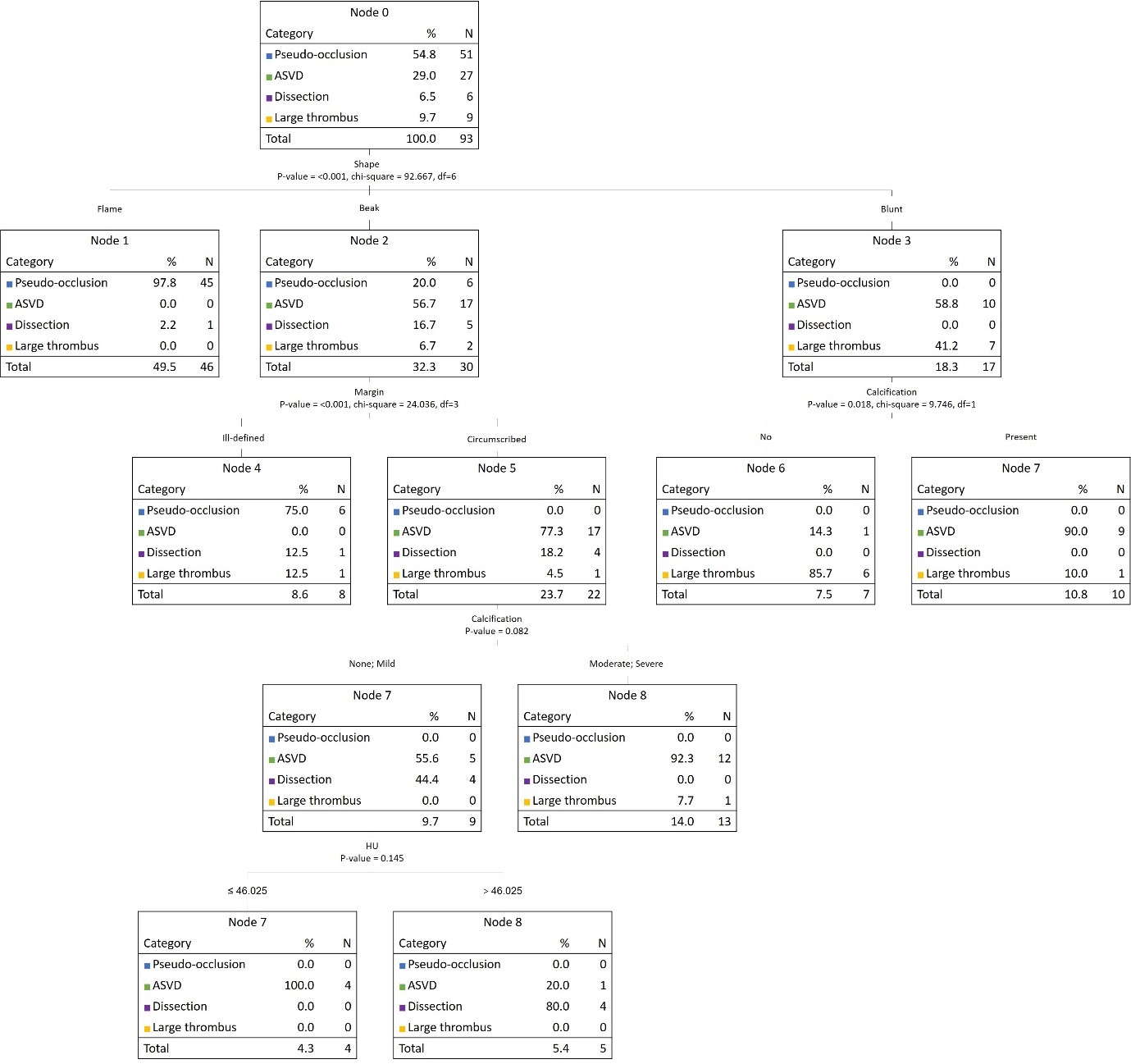

Supplement: Supplementary file 1 [file diagnostics-14-01524-s001.zip › Figure S8_Diagnostic tree.jpg]

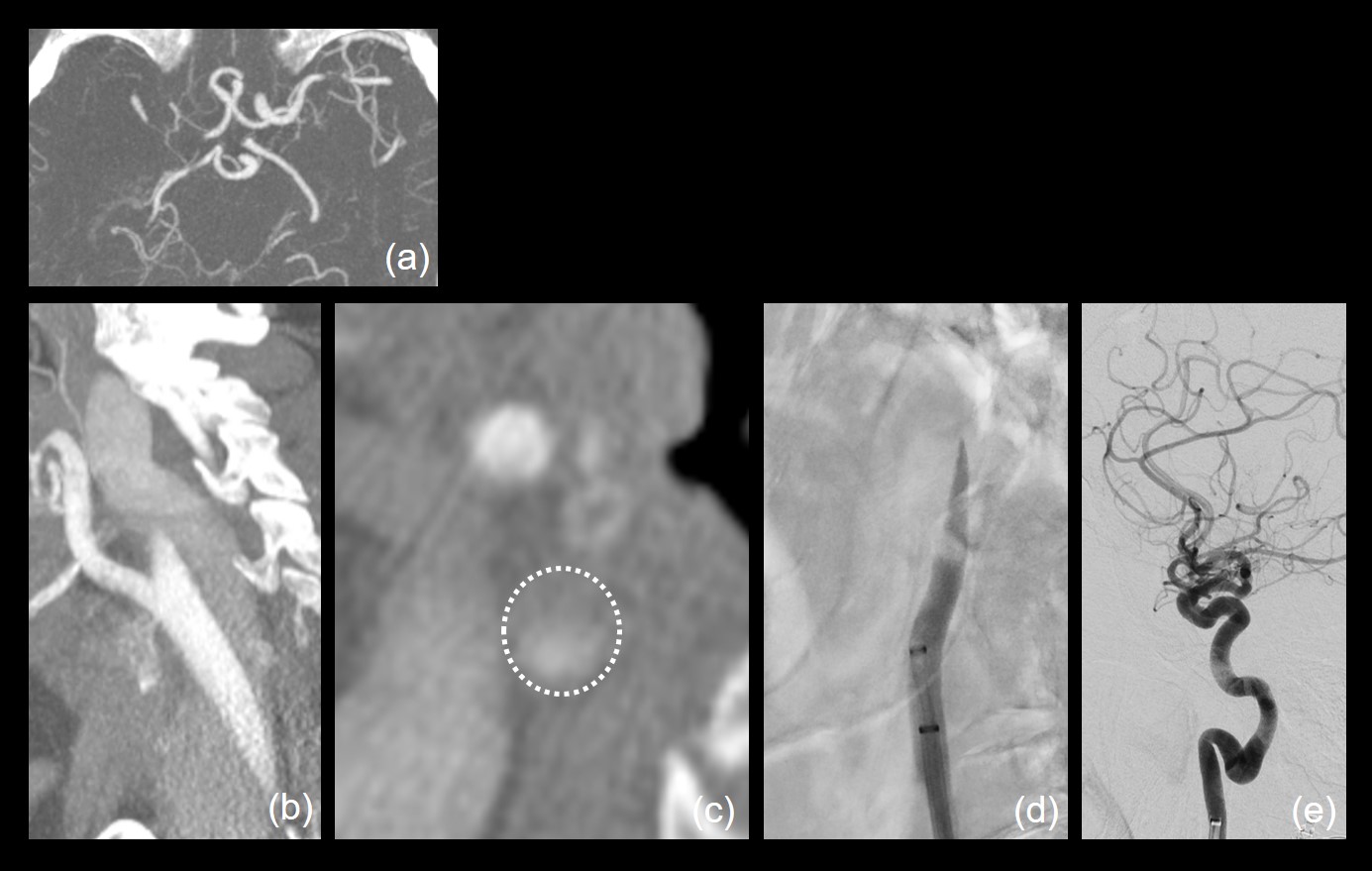

Supplement: Supplementary file 1 [file diagnostics-14-01524-s001.zip › Figure S9_Kinking case (a-e).jpg]
